# Clinical recognition of frontotemporal dementia with right anterior temporal predominance: A multicenter retrospective cohort study

**DOI:** 10.1002/alz.14076

**Published:** 2024-07-10

**Authors:** Hulya Ulugut, Maxime Bertoux, Kyan Younes, Maxime Montembeault, Giorgio G. Fumagalli, Bedia Samanci, Ignacio Illán‐Gala, Gregory Kuchcinski, Melanie Leroy, Jennifer C. Thompson, Christopher Kobylecki, Alexander F Santillo, Elisabet Englund, Maria Landqvist Waldö, Lina Riedl, Jan Van den Stock, Mathieu Vandenbulcke, Rik Vandenberghe, Robert Laforce Jr, Simon Ducharme, Peter S. Pressman, Paulo Caramelli, Leonardo Cruz de Souza, Leonel T. Takada, Hakan Gurvit, Oskar Hansson, Janine Diehl‐Schmid, Daniela Galimberti, Florence Pasquier, Bruce L. Miller, Philip Scheltens, Rik Ossenkoppele, Wiesje M. van der Flier, Frederik Barkhof, Nick C. Fox, Virginia E. Sturm, Toji Miyagawa, Jennifer L. Whitwell, Bradley Boeve, Jonathan D. Rohrer, Maria Luisa Gorno‐Tempini, Keith A. Josephs, Julie Snowden, Jason D. Warren, Katherine P. Rankin, Yolande A. L. Pijnenburg, International rtvFTD Working Group

**Affiliations:** ^1^ Alzheimer Center Amsterdam Department of Neurology Amsterdam Neuroscience Vrije Universiteit Amsterdam Amsterdam UMC De Boelelaan Amsterdam The Netherlands; ^2^ Memory and Aging Center Department of Neurology UCSF Weill Institute for Neurosciences University of California San Francisco California USA; ^3^ Lille Neuroscience & Cognition U1172, Univ. Lille, Inserm, CHU Lille, LiCEND & Labex DistALZ Lille France; ^4^ Stanford Neuroscience Health Center Department of Neurology Stanford University Palo Alto California USA; ^5^ Department of Psychiatry Douglas Mental Health University Institute McGill University Health Centre McGill University Montreal Quebec Canada; ^6^ Department of Neurology University of Milan Milan Italy; ^7^ Università degli Studi di Trento | UNITN·CIMEC ‐ Center for Mind/Brain Sciences Mattarello Trentino Italy; ^8^ Department of Neurology Istanbul University Fatih Istanbul Turkey; ^9^ Sant Pau Memory Unit Department of Neurology Hospital de la Santa Creu i Sant Pau Biomedical Research Institute Sant Pau Universitat Autònoma de Barcelona Barcelona Spain; ^10^ Centro de Investigación en Red‐Enfermedades Neurodegenerativas (CIBERNED) Madrid Spain; ^11^ Cerebral Function Unit, Greater Manchester Neuroscience Centre Salford Royal NHS Foundation Trust Salford UK; ^12^ Division of Neuroscience and Experimental Psychology Faculty of Biology Medicine and Health University of Manchester Salford Manchester UK; ^13^ Department of Neurology Manchester Centre for Clinical Neurosciences NHS Foundation Trust Salford UK; ^14^ Division of Neuroscience University of Manchester Salford Manchester UK; ^15^ Clinical Memory Research Unit Department of Clinical Sciences Faculty of Medicine Lund University Lund Sweden; ^16^ Division of Pathology Department of Clinical Sciences Lund University Lund Sweden; ^17^ Division of Clinical Sciences Helsingborg Department of Clinical Sciences Lund Lund University Lund Sweden; ^18^ School of Medicine Department of Psychiatry and Psychotherapy Technical University of Munich Munich Germany; ^19^ Neuropsychiatry, Department of Neurosciences Leuven Brain Institute Leuven Belgium; ^20^ Department of Neurology University Hospital Leuven Leuven Belgium; ^21^ Clinique Interdisciplinaire de Mémoire (CIME) Département des Sciences Neurologiques Laval University Quebec City Canada; ^22^ Anschutz Medical Campus Behavioral Neurology Section Department of Neurology University of Colorado Aurora Colorado USA; ^23^ Behavioral and Cognitive Neurology Unit Department of Internal Medicine Faculdade de Medicina Universidade Federal de Minas Gerais Belo Horizonte Brazil; ^24^ Cognitive and Behavioral Unit Hospital das Clinicas Department of Neurology University of São Paulo Medical School Pacaembu São Paulo Brazil; ^25^ Kbo‐Inn‐Salzach‐Klinikum Clinical Center for Psychiatry Psychotherapy, Psychosomatic Medicine, Geriatrics and Neurology Wasserburg/Inn Germany; ^26^ Department of Biomedical Surgical and Dental Sciences University of Milan Milan Italy; ^27^ Fondazione IRCCS Ca’ Granda Ospedale Maggiore Policlinico Milan Italy; ^28^ Alzheimer Center Amsterdam Department of Radiology Amsterdam Neuroscience Vrije Universiteit Amsterdam Amsterdam UMC De Boelelaan Amsterdam The Netherlands; ^29^ UCL Institutes of Neurology and Healthcare Engineering University College London London UK; ^30^ Dementia Research Centre UCL Queen Square Institute of Neurology London UK; ^31^ Department of Neurology Mayo Clinic, Rochester Rochester Minnesota USA; ^32^ Department of Radiology Mayo Clinic, Rochester Rochester Minnesota USA; ^33^ Dyslexia Center University of California San Francisco UCSF Weill Institute for Neurosciences University of California San Francisco California USA

**Keywords:** emotion recognition, frontotemporal dementia, frontotemporal lobar degeneration, right anterior temporal lobe, semantic dementia, social cognition

## Abstract

**INTRODUCTION:**

Although frontotemporal dementia (FTD) with right anterior temporal lobe (RATL) predominance has been recognized, a uniform description of the syndrome is still missing. This multicenter study aims to establish a cohesive clinical phenotype.

**METHODS:**

Retrospective clinical data from 18 centers across 12 countries yielded 360 FTD patients with predominant RATL atrophy through initial neuroimaging assessments.

**RESULTS:**

Common symptoms included mental rigidity/preoccupations (78%), disinhibition/socially inappropriate behavior (74%), naming/word‐finding difficulties (70%), memory deficits (67%), apathy (65%), loss of empathy (65%), and face‐recognition deficits (60%). Real‐life examples unveiled impairments regarding landmarks, smells, sounds, tastes, and bodily sensations (74%). Cognitive test scores indicated deficits in emotion, people, social interactions, and visual semantics however, lacked objective assessments for mental rigidity and preoccupations.

**DISCUSSION:**

This study cumulates the largest RATL cohort unveiling unique RATL symptoms subdued in prior diagnostic guidelines. Our novel approach, combining real‐life examples with cognitive tests, offers clinicians a comprehensive toolkit for managing these patients.

**Highlights:**

- This project is the first international collaboration and largest reported cohort.
- Further efforts are warranted for precise nomenclature reflecting neural mechanisms.
- Our results will serve as a clinical guideline for early and accurate diagnoses.

## 1 BACKGROUND

Frontotemporal dementia (FTD) is a neurodegenerative disorder that predominantly affects the frontal and/or temporal lobes and presents with a spectrum of social, behavioral, language, psychiatric, and motor problems.^1^, ^2^, ^3^ Its clinical presentation varies depending on the predominance of the affected regions. Based on the latest diagnostic criteria, social and personal behavioral features are covered in the consensus criteria for behavioral variant FTD (bvFTD),^2^ whereas language variants are clustered under the term primary progressive aphasia (PPA), namely the semantic variant (svPPA), the non‐fluent variant (nfvPPA), and the logopenic variant (lvPPA), although the latter has most often been underlying Alzheimer's disease (AD) pathology.^3^ The bvFTD has been associated with symmetric or asymmetric atrophy of the frontal and/or temporal lobes; nfvPPA with left peri‐sylvian involvement, and svPPA with predominant involvement of the left anterior temporal lobe. A separate variant with predominant right anterior temporal lobe (RATL) atrophy has been recognized, and labeled «right temporal variant FTD», «right‐lateralized SD», «right temporal lobe atrophy», «right temporal variant PPA», «right sided svPPA», and most recently «semantic behavioral variant FTD»,^3^, ^4^, ^5^, ^6^, ^7^, ^8^, ^9^ without consensus on its accompanying clinical syndrome.^10^

However, the diagnostic criteria for svPPA and earlier criteria for FTD and semantic dementia (SD) allude to RATL involvement. Semantic deficits for people and objects across various sensory modalities, loss of empathy, and compulsions were mentioned in the criteria for svPPA as characteristics of RATL predominant svPPA,^3^ and hypochondriasis, evanescent bizarre somatic preoccupations, prosopagnosia, associative agnosia, loss of sympathy and empathy, and parsimony were listed in the first research and clinical diagnostic criteria for FTD that might refer to RATL involvement.^1^, ^11^

In several articles, non‐verbal, mainly visual, semantic deficits have been underscored,^6^, ^12^ and several unique symptoms such as person specific semantic loss, hyper‐religiosity, somatization, topographagnosia, delusions, emotional coldness, and depression have been associated with the RATL,^4^, ^5^, ^7^, ^8^, ^9^ although different clinical terminologies have been used for clinical symptoms and discrepant results have been published regarding the clinical characteristics and nature of the syndrome.^4^, ^5^, ^6^, ^7^, ^8^, ^9^ One of the explanations for the discrepancies in reported clinical characteristics of the syndrome is the use of different clinical terminologies, within the respective fields of neurology and psychiatry, or between them.^4^, ^5^, ^6^, ^7^, ^8^, ^9^ For instance, some groups have interpreted behavioral symptoms through a semantic deficit perspective, emphasizing deficits in socioemotional semantics.^9^, ^13^ In contrast, other groups have employed social cognition terminologies, such as emotion recognition, mentalizing, and valence (hedonic),^5^, ^14^ while yet others have used broader terms like lack of empathy,^7^ or psychiatric terms like alexithymia.^15^ Therefore, not surprisingly, different frameworks have been proposed to recognize the syndrome at early stages. A large clinical study has reported behavioral changes in 95%, episodic memory impairment in 60%, and prosopagnosia in 54% of amyloid negative FTD patients with RATL atrophy, while depression, somatic complaints, and motor/mental slowness have been the most distinctive symptoms compared to svPPA, bvFTD, and AD.^7^ More recently, Younes et al., described the characteristics of their cohort by using objective atrophy rating and novel cognitive assessments measuring semantic knowledge and social cognition. Loss of empathy, loss of person‐specific knowledge, and complex compulsive behavior/mental rigidity have been described as the most characteristic and presenting hallmarks of the syndrome, although being less prevalent (27%, 23%, and 18%, respectively).^9^ These single‐center studies illustrate the need for a common nomenclature and consensus diagnostic criteria based on multicultural data.

Therefore, in 2020, we established an international working group (IWG) that aims to improve the recognition of FTD with predominant RATL involvement in daily clinical practice. We set out to (i) systematically collect multicenter retrospective data to generate a set of the most relevant clinical features, and (ii) tease out the interpretation of the individual symptoms as well as tools to detect and monitor them, by the IWG in round table meetings. This present work focuses on the initial objective, encompassing the collation of retrospective clinical data from 18 specialized dementia centers (Table 1).

**TABLE 1 alz14076-tbl-0001:** List of the centers (based on the sample size)

	Center (sample size)
1	Amsterdam (*n* = 82)
2	San Francisco (*n* = 47)
3	London (*n* = 34)
4	Rochester (*n* = 27)
5	Manchester (*n* = 23)
6	Lille (*n* = 23)
7	Lund Malmo (*n* = 22)
8	Lund LUPROFS (*n* = 16)
9	Istanbul (*n* = 15)
10	Milan (*n* = 15)
11	Barcelona (*n* = 12)
12	Leuven (*n* = 11)
13	Munich (*n* = 9)
14	Quebec (*n* = 8)
15	Montreal (*n* = 5)
16	Colorado (*n* = 4)
17	Brazil USP (*n* = 4)
18	Brazil Belo Horizonte (*n* = 3)

Since many aspects of RATL degeneration wait to be tested and there is no consensus on nomenclature yet, in this article, we use the term rtvFTD (right temporal variant of FTD) to bracket our inclusion criteria: patients with (i) a clinical diagnosis of any form of FTD, and (ii) RATL atrophy on structural neuroimaging at clinical presentation.

## 2 METHODS

Details of the establishment of the IWG, consensus approach, list of members, dates and agenda of each round table meeting, the roadmap of the IWG, patient selection, data collection, approach on missing data, list of the available cognitive tests, harmonization, and analysis of the data can be found in the supplementary materials.

**RESEARCH IN CONTEXT**

1. **Systematic review**: A comprehensive review in MEDLINE (PubMed) and Embase covering the literature in the English language until March 2023 was performed. The search terms included “frontotemporal dementia AND right temporal,” “semantic dementia,” “semantic variant primary progressive aphasia AND right,” “behavioral variant frontotemporal dementia AND right,” “temporal variant frontotemporal dementia,” and “frontotemporal lobar degeneration AND right temporal.”
2. **Interpretation**: Diverse and sometimes conflicting descriptions have characterized the symptoms attributed to frontotemporal dementia (FTD) with right anterior temporal lobe (RATL) predominance. In this largest reported multicultural cohort study by an international working group, we provide real‐world examples drawn from case reports, common symptomatology, accompanied by cognitive test outcomes offering guidance to clinicians in identifying and subsequently directing these patients to specialized dementia centers.
3. **Future directions**: An urgent need in the field is consensus on terminologies on FTD with RATL degeneration that elucidate primary cortical dysfunctions and can be readily applied in everyday clinical practice.

### 2.1 Data collection

In 2020, a dataset template was prepared based on the published RATL degeneration/temporal variant FTD literature and included (1) demographic; (2) clinical data (including symptoms, family history, dementia severity, and cognitive and neuropsychiatric tests); (3) atrophy rating scores for each anatomical area; (4) biomarkers (cerebrospinal fluid [CSF] amyloid, tau, amyloid positron emission tomography [PET], neurofilament light chain [NfL], or other potential biomarkers for FTD‐if available); (5) genetic mutations; (6) pathological information. A broad range of clinical symptoms (cognitive, behavioral, language, psychiatric, and other) were coded as present, absent, or not available at the initial and follow‐up visits. If a symptom was present, the collectors were asked to describe the symptom by recording real‐life examples from the case notes. Additionally, there was an “other” column for each section where the collectors were able to add original data/information that was not on the predefined list. Due to the diversity in missing data, the number of available data for each symptom is displayed alongside the corresponding percentage in the results, for further clarification. All cases had symptom checklist charts generated by the IWG and nearly half of the sample had details of the case notes (real‐life examples) for each symptom (*n* = 134). Data collection was completed in July 2022.

### 2.2 Patient selection

Among subjects[Table alz14076-tbl-0001] registered with any clinical form of FTD, those who demonstrated predominant RATL atrophy on the initial neuroimaging were selected. For identifying the RATL atrophy pattern, visual rating scales^16^ were used, and if the atrophy on the RATL was higher than the non‐RATL areas (left temporal or frontal), this patient was considered as a case of rtvFTD. Patients with frontal/left temporal atrophy ≥3 (max score = 4) (even if they had predominant RATL atrophy) were excluded to minimize the gross effects of general neurodegeneration. This selection resulted in four patients with a non‐RATL areas atrophy score of 0, 116 patients with a score of 1, and 240 patients with a score of 2.

### 2.3 Analysis

Variables were reported as means and standard deviations or proportions when appropriate. The frequency of individual symptoms was reported as proportions. Frequencies were calculated based on the available data, excluding any missing data. Symptoms were listed based on their presence at the initial visit as a categorical variable and frequencies were calculated. Subsequently, each reported symptom was associated with real‐life examples and cognitive tests if available, to better describe the nature of the symptom. Continuous variables like cognitive test scores were displayed by harmonizing Z scores. Z scores were calculated for each cognitive test and each participant (supplementary material).

### Data availability

Signed data sharing agreements between centers do not permit open data sharing.

## 3 RESULTS

Table 2 displays the total sample size (*n* = 360), demographic data, symptom duration, follow‐up duration, handedness, available neuroimaging, and biomarkers (amyloid, NfL) data, initial Clinical Dementia Rating (CDR) and Mini‐Mental State Examination (MMSE) scores of the patient group. The pathology findings, genetic risk factors, biomarker profile, and the impact of amyloid positivity will be presented in a separate publication by the IWG.

**TABLE 2:** Characteristics of the collected data

Parameter	Total sample
*N* (total), *N* ^a^ (definite)	360, 67
Sex, *n* female (%)	156 (43%)
Age, years, mean ± SD	65,9 ± 8,6
Handedness: right/left/ambidexterous/unknown	316/18/7/19
Symptom duration, years, mean ± SD	3.5 ± 2.1
Follow‐up period, years, median (min–max)	9.3, (0–18)
CDR initial visit, n, mean ± SD	*n* = 241 (1.2 ± 1)
MMSE initial visit, median (min–max)	*n* = 316 (18, 7–30)
Neuroimaging available	MRI *n* = 343, CT *n* = 19, PET (amyloid) *n* = 21, PET (FDG) *n* = 16, SPECT *n* = 1
Biomarker	CSF (amyloid, tau, phospho tau) *n* = 211, NfL *n* = 43

Abbreviations: CDR, Clinical Dementia Rating; MMSE, Mini‐Mental State Examination; MRI, magnetic resonance imaging; CT, computer tomography; PET, positron emission tomography; FDG, fluorodeoxyglucose; SPECT, single photon emission computed tomography; CSF, cerebrospinal fluid; NfL, neurofilament light chain.

^a^ Some subjects have both genetic and pathological confirmation.

### 3.1 Clinical profile

Our approach was twofold. First, we identified the most common retrospectively reported symptoms, based on a predefined symptom checklist with room to report additional symptoms not mentioned in the list. Next, we associated symptoms with available cognitive test results (i.e., the association between reported memory problems and the results of memory tests). Also, we placed reported symptoms into context by using the examples that were provided in the case notes (i.e., describing under which circumstances a patient was forgetful).

#### 3.1.1 Clinical profile based on the chart reviews

**FIGURE 1:**
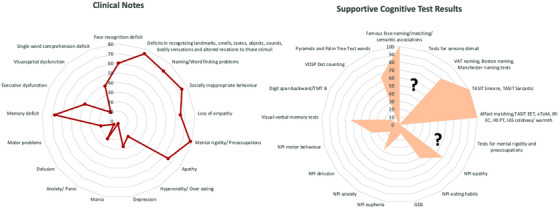
Representation of the collected clinical data. The red line represents clinical symptoms, while the orange shadows depict associated cognitive test results available in the collected dataset. Cognitive tests and behavioral assessments were limited to smells, tastes, sounds, bodily sensations, objects, landmarks, mental rigidity, and preoccupations, which are denoted by a question mark (?) in the figure

Analysis of the historical case notes showed that the most often reported symptoms at the initial visit were compulsive behavior (78%), disinhibition/socially inappropriate behavior (74%), naming/word finding difficulties (70%), memory deficit (67%), apathy (65%), loss of empathy (65%), and face recognition deficit (60%) (Figure 1). It is worth noting that the aforementioned symptoms had been systematically collected by dementia centers over the years as part of already published diagnostic criteria for FTD, SD, bvFTD, PPA, or AD. Additionally, to avoid any potential bias, the top‐rated symptoms from five centers with the largest sample sizes were compared, and similar distributions were noted (Supplementary Material).

Moreover, a sub‐analysis was conducted on cases scored as “0: not impaired” in non‐RATL areas (left temporal and bilateral frontal regions). Among these patients (*n* = 4), symptom distributions were observed, revealing apathy/social withdrawal (*n* = 3), socially inappropriate behavior (*n* = 3), mental rigidity (*n* = 3), preoccupations (*n* = 3), loss of empathy (*n* = 2), memory deficit (*n* = 2), face recognition deficit (*n* = 2), place/landmark recognition deficit (*n* = 1), taste recognition deficit (*n* = 1), word finding and naming problems (*n* = 2), depression (*n* = 2), and slowness (*n* = 1).

**FIGURE 2:**
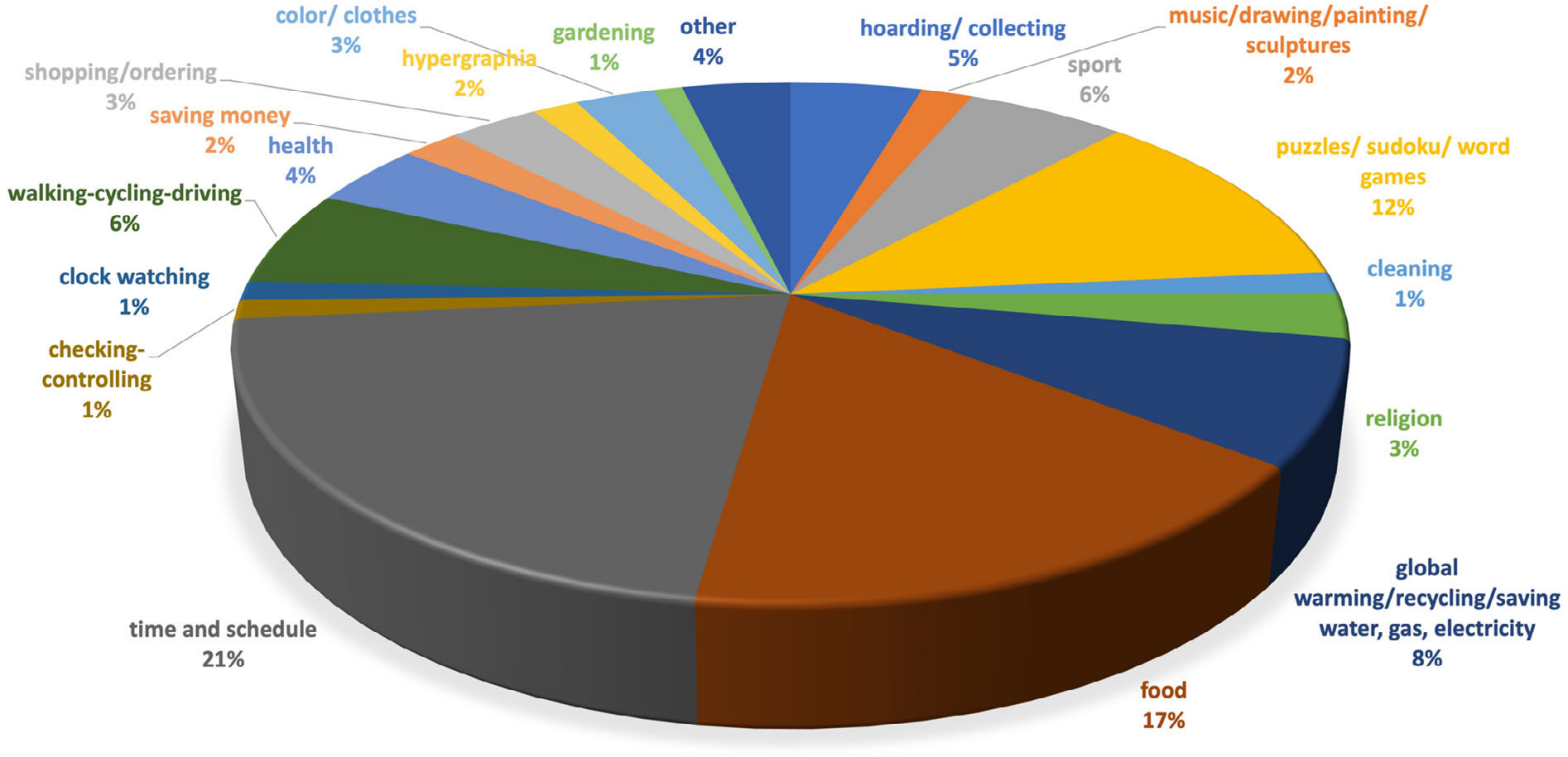
Reported specific interests. One patient may have more than one symptom

For compulsive behavior, mental rigidity and/or preoccupations were specifically mentioned in all cases as a subcategory of our symptom checklist. Furthermore, in total, 505 specific examples were reported for specific interests: time and schedule (21%), food (17%), puzzles/sudoku/computer games (12%), global warming/recycling/saving gas‐water‐electricity (8%), sports (6%), walking/cycling/driving (6%), hoarding/collecting (5%), health‐related (4%), shopping/ordering (3%), colors (3%), clothes (3%), religion (3%), writing (2%), art (2%), saving money/parsimony (2%), cleaning (1%), clock watching (1%), checking/controlling (1%), gardening (1%), and other (4%) (Figure 2).

Additionally, out of the total 360 patients, data for psychiatric symptoms were available for 291 patients (reported as present or absent), with 69 patients' data being “missing.” Among these 291 patients, 166 had psychiatric manifestations (57%) such as affective dysregulation (39%), delusions/hallucinations (i.e., hearing God's voice, seeing deceased mother, etc.) (14%), and anxiety/panic (35%). Besides psychiatric symptoms, word comprehension deficits in 39% patients (available *n* = 360), object recognition deficits in 25% (available *n* = 311), and motor problems (pyramidal or extrapyramidal) in 19% (available *n* = 360) were reported. As additional symptoms, they exhibited recognition problems and altered reactions to the following stimuli (available *n* = 239): taste (45%), landmarks (37%), bodily sensations (28%), smell (2%), and sound (2%). When those domains were clustered, it was found that at least one of these symptoms was present in 74% of the patients. Of note, different terminologies were used for those symptoms (i.e., for landmarks: “topographagnosia,” “problems with naming landmarks,” “cannot recognize buildings”; for taste: “gustatory agnosia,” “cannot differentiate the taste,” “flavor recognition problem,” etc.), and they were mentioned in different symptom categories by different data collectors (i.e., whereas “deficit in taste” was mostly reported in the language or behavioral category, and “deficit in bodily sensations” in the psychiatric category as “somatization” or in other as “alexisomia,” landmarks were usually mentioned under the symptom subheading “getting lost” or “naming difficulties” or “word finding difficulties” or “other”).

Last, orientation problems, apraxia, calculation problems, visuospatial problems, concentration problems, agraphia, alexia, hypersexuality, hyperorality, and utilization were the least rated symptoms (less than 15%), although they were in the symptom checklist in the chart reviews.

#### 3.1.2 Clinical profile based on the combination of chart reviews and available case note details and cognitive test results

When possible, each symptom was associated with real‐life examples from case notes and relevant cognitive test results (Figure 1, Tables 3 and 4). The number of available data for each cognitive test can be found in Table 3.

**TABLE 3:** Cognitive test scores

Parameter	Test (max score)	*N*	Mean ± SD	Norm scores	*Z* scores (mean)	Impaired/affected (individual)^a^
Visual memory	Visual association task (VAT) (max = 6)	84	4.66 ± 1.73	5.41 ± 0.99^(^ ^1^ ^)^	−0.75	21%
	Benson complex figure–delay (max = 17)	44	5.6 ± 0.5	11.2 ± 3.2^(^ ^2^ ^)^	−7.9	57%
	Visual Object Memory Test (delayed recall)^d^	20	2.78 ± 3.47	9.7 ± 2.9^(^ ^3^ ^)^	−2.38	82%
Verbal memory	Rey Auditory Verbal Learning Test (RAVLT) recall	85	5 ± 3.7	10.5 ± 3.3^(^ ^4^ ^)^	−1.67	57%
	California Verbal Learning Test (CVLT) recall	43	3.9 ± 0.2	11.6 ± 2.9 ^(^ ^5^ ^)^	−2.7	62%
	ADAS‐3 delayed recall (max = 10)	21	3.2 ± 2.6	3.82 ± 2.15^(^ ^6^ ^)^	−0.33	22%
Memory other	WMS‐R logical memory delayed	5	1.2 ± 2.1	12.5 ± 3.9^b^ ^,(^ ^7^ ^)^	−2.89	100%
	Short story (max = 16)	7	3.6 ± 2.6	Cut‐off 4.5		57%
Verbal semantic	Manchester naming test	23	29.8 ± 7.9	^b^	−4.74	65%
	Graded Naming test	23	4.57 ± 4.94	20.4 ± 4.1^(^ ^8^ ^)^	−3.86	87%
	Boston Naming Test (long) (max = 60)	114	33.9 ± 16.6	55.3 ± 3.7^(^ ^9^ ^)^	−5.77	79%
	Pyramids and Palm Trees/words (max = 52)	46	32.4 ± 14.47	49.44 ± 1.90^(^ ^10^ ^)^ Cut off = 47/52 ^(^ ^11^ ^)^	−3.99	65%
	VAT Naming (max = 12)	73	10.2 ± 3.3	11.89 ± 1.1^b^	−1.53	50%
Visual semantic	Pyramids and Palm Trees/pictures (max = 52)	33	41.8 ± 2.91	49.44 ± 1.90^(^ ^10^ ^)^	−3.85	96%
	CATS Face Matching (max = 12)	31	11.24 ± 0.2	11.55 ± 0.18^b^	−1.72	48%
	Famous Faces Naming (max = 20)	15	1.26 ± 0.86	12.82 ± 0.87^b^	−13.32	100%
	Famous Faces Familiarity (max = 20)	24	6.85 ± 0.88	14.19 ± 1.10^b^	−6.46	91%
	Famous Faces Semantic Association (max = 20)	12	5.37 ± 1.13	14.71 ± 0.96^b^	−10.70	100%
	Famous Faces Name Familiarity (max = 20)	14	2.80 ± 0.78	11.91 ± 0.94^b^	−10.54	100%
Social function and emotion	CATS Affect Matching (max 16)	35	9.11 ± 0.42	12.82 ± 0.46^b^	−8.02	88%
	TASIT EET (max = 14)	27	6.46 ± 0.48	10.89 ± 0.40^b^	−10.74	92%
	TASIT SI‐M Sincere (max 20)	24	15.99 ± 0.69	16.69 ± 0.55^b^	−2.12	36%
	TASIT SI‐M Sarcastic (max = 20)	24	4.74 ± 0.85	17.60 ± 0.68^b^	−19	100%
	IRI Empathetic Concern (max = 24)	44	16.09 ± 1.41	27.41 ± 1.56^b^	−7.38	84%
	IRI Perspective Taking (max = 24)	44	10.77 ± 1.10	22.86 ± 1.22^b^	−10.13	93%
	Emotional Theory of Mind (max = 16)	9	12.25 ± 0.46	14.62 ± 0.35^b^	−6.43	100%
	Cognitive Theory of Mind (max = 16)	15	14.79 ± 0.58	15.07 ± 0.35^b^	−0.77	33%
	IAS‐Current Warmth	13	37.59 ± 3.02	47.89 ± 2.12^b^	−5.13	61%
	IAS‐Current Coldness	13	29.53 ± 2.27	13.72 ± 1.83^b^	8.86	89%
Language	Animal fluency (max = 49)	180	11.7 ± 5.7	21.4 ± 5.7^(^ ^2^ ^)^	−1.69	60%
	Lexical fluency	43	8.53 ± 0.64	14.09 ± 0.81^b^	−6.82	50%
	Letter fluency (FAS)	22	18.4 ± 10.1	^b^		26%
Executive functioning	Digit span backward (longest span, max = 14)	198	6.7 ± 3.2	7.2 ± 2.2^(^ ^2^ ^)^	−0.19	12%
	TMT‐B (seconds, 13‐300^c^)	122	121.95 ± 62.05	82.2 ± 46.3^(^ ^2^ ^)^	0.85	14%
Attention	Digit span forward (longest span, max = 9)	171	5.39 ± 1.5	6.7 ± 1.3^(^ ^2^ ^)^	−1	33%
	TMT‐A (seconds, 13–150^c^)	166	57.11 ± 27.39	30.9 ± 13.4^(^ ^2^ ^)^	1.95	37%
Visuospatial functioning	Incomplete letters (VOSP)	81	17.2 ± 4.3	19.46 ± 0.73^(^ ^12^ ^)^	−0.73	38%
	Dot counting (VOSP)	41	9.7 ± 1.1	9.68 ± 0.68^(^ ^12^ ^)^	0.11	2%
Depression	Geriatric depression scale (GDS)	69	4.2 ± 4.6	normal‐0‐9	–	11% mild depressive, no severe depression
				mild depressive‐10‐19		
				severe depressive‐20‐30.^(^ ^13^ ^)^		

^a^ The percentages of the patients whose z score is lower than −1.67.

^b^ Age and sex matched healthy control.

^c^ Default time when the test is not completed.

^d^ A multi‐modal task involving delayed verbal recall of objects visually presented to and named by participants in the learning phase.

REFERENCES

^1^ Lindeboom J, Schmand B, Tulner L, Walstra G, Jonker C. Visual association test to detect early dementia of the Alzheimer type. J Neurol Neurosurg Psychiatry. 2002 Aug;73(2):126‐33.

^2^ Weintraub S, Besser L, Dodge HH, Teylan M, Ferris S, Goldstein FC, et al. Version 3 of the Alzheimer Disease Centers’ Neuropsychological Test Battery in the Uniform Data Set (UDS). Alzheimer Dis Assoc Disord. 2018 Mar;32(1):10‐7.

^3^ Stopford, CL, Snowden, JS, Thompson, JC, Neary, D. (2007). Distinct memory profiles in Alzheimer's disease. *Cortex*. *43*, 846‐857.

^4^ Loring DW, Saurman JL, John SE, Bowden SC, Lah JJ, Goldstein FC. The Rey Auditory Verbal Learning Test: Cross‐validation of Mayo Normative Studies (MNS) demographically corrected norms with confidence interval estimates. J Int Neuropsychol Soc. 2022 Apr 28;1‐9.

^5^ Kramer AO, Casaletto KB, Umlauf A, Staffaroni AM, Fox E, You M, et al. Robust normative standards for the California Verbal Learning Test (CVLT) ages 60‐89: A tool for early detection of memory impairment. Clin Neuropsychol. 2020 Feb;34(2):384‐405.

^6^ Sano M, Raman R, Emond J, Thomas RG, Petersen R, Schneider LS, et al. Adding Delayed Recall to the Alzheimer Disease Assessment Scale is Useful in Studies of Mild Cognitive Impairment but Not Alzheimer Disease. Alzheimer Dis Assoc Disord. 2011;25(2):122‐7.

^7^ Montgomery V, Harris K, Stabler A, Lu LH. Effects of Delay Duration on the WMS Logical Memory Performance of Older Adults with Probable Alzheimer's Disease, Probable Vascular Dementia, and Normal Cognition. Arch Clin Neuropsychol. 2017 May;32(3):375‐80.

^8^ Warrington, EK (1997) Graded Naming Test: a restandardisation. Neuropsychological Rehabilitation. 7(2), 143‐146.

^9^ Zec RF, Burkett NR, Markwell SJ, Larsen DL. Normative data stratified for age, education, and gender on the Boston Naming Test. Clin Neuropsychol. 2007 Jul;21(4):617‐37.

^10^ Callahan BL, Macoir J, Hudon C, Bier N, Chouinard N, Cossette–Harvey M, et al. Normative Data for the Pyramids and Palm Trees Test in the Quebec‐French Population. Arch Clin Neuropsychol. 2010 May 1;25(3):212‐7.

^11^ Howard D, Patterson KE. The Pyramids and Palm Trees Test. https://eprints.ncl.ac.uk [Internet]. 1992 [cited 2022 Sep 30]; Available from: https://eprints.ncl.ac.uk.

^12^ Bonello PJ, Rapport LJ, Millis SR. Psychometric properties of the visual object and space perception battery in normal older adults. Clin Neuropsychol. 1997 Nov 1;11(4):436‐42.

^13^ Brink TL, Yesavage JA, Lum O, Heersema PH, Adey M, Rose TL. Screening Tests for Geriatric Depression. Clin Gerontol. 1982 Oct 14;1(1):37‐43.

**TABLE 4:** Summary of the overall results and interpretation

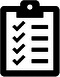	Chart review	Real‐life examples	Available cognitive tests	Interpretation/future directions
**B** **L** **M**	Face recognition deficit	*“…she cannot recognize us anymore…”* *“…he is struggling with identifying famous people while we are watching TV…”*	Impairment in face matching, face familiarity, famous face semantic association, and famous face name familiarity.	Deficit is not limited to the visual perception of a face. The term defining the deficit should be reconsidered
**B** **L** **M**	Recognition difficulties and altered reactions to sound, smell, taste, landmarks, bodily sensations	*“…he drank from a bottle of soap, confusing it with food… He cannot differentiate the taste of soap or wine anyway…”* *“…little sense of smell, ignoring the strong smell of a skunk in their area…”* *“…she cannot recognize many kitchen tools and food, she stopped cooking…”* *“…he overreacts to innocuous physical stimuli…”*	Not available	Available data are not sufficient. Further efforts are warranted to encapsulate such deficits.
**L**	Naming/word finding difficulties	*“…finding the names for example family members, food, well known places is not easy anymore…”*	Results varied between 50% and 87% across five different naming tests	Divergent naming test outcomes and the nature of real‐life examples warrant further attention to this symptom
**M**	Memory deficit	*“…difficulties with remembering people…”* *“…asking repetitive questions as well as having a lot of difficulty with recent events, sometimes forgets appointments…”* *“…he can remember what he had for breakfast, but he has no recollection of either the events of September 11, 2001, or of the recent tsunami in Southeast Asia…”* *“…she is losing the emotional aspects in memories. She can describe how she worked on Robert Kennedy's political campaign, but she cannot give details about her feelings when he or John F Kennedy were killed…”*	Sample sizes are too small to interpret logical memory and craft story delayed scores. The greatest impairment was observed in the visual object memory test (87%). Results varied between 21% and 57% across two other visual memory tests, and between 22% and 62% across three verbal memory tests	Divergent memory test outcomes and the nature of real‐life examples warrant further attention to this symptom
**M** **B**	Lack of empathy	*“…the wife noted that the patient has been less sensitive to recognize emotions. For instance, she was crying after the Virginia Tech tragedy, and he could not understand why she was sad and proceeded to ask for a ham sandwich…”* *“…he has become very cold and does not show any emotions. He does not seem to mind or notice when his wife is upset …”*	Impairment in emotion recognition and emotional theory of mind, however, better performances in cognitive theory of mind. Informant questionnaires indicated lower empathic concern, perspective taking, and prominent interpersonal coldness.	The term defining the deficit should be specified.
**B**	Apathy	*“…less enthusiastic for certain things such as meeting up friends, family members…”* *“…socially withdrawn…”* *“…she does not socialize much with friends anymore…”* *“…no more interest previous hobbies. She left all social clubs even if she was the president of the book club, and vice president of the gardening club. She prefers gardening by herself…”*	Apathy was reported in 69% of the tested participants by informants in the Neuropsychiatric Inventory	Real life examples differ the symptom from classical cognitive apathy. The term defining the deficit should be specified.
**B**	Disinhibition/socially inappropriate behavior	*“…He cannot read the body language and easily believe what people say… she purchased a vacuum cleaner from a door‐to‐door salesman… became more gullible”* *“…She has developed some loss of concern over personal boundaries, and is frequently bumping into people and staring almost inappropriately at strangers (to see if she recognizes them)…”* *“…He decided to be the golf champion in the Netherlands and spent his entire time and money for this sport, even though he became extremely stingy regarding other daily life activities, including costs for showering…”* *“… he gets easily aggressive if we don't follow his schedule…”*	Impairment in sarcasm detection whereas better performances in comprehension of sincere social interaction.	Available tests are not sufficient to explain real life examples. Further work is needed in this domain and the term defining the deficit should be specified.
**B**	Mental rigidity and preoccupations	*“… he is very tight in time and schedule… he gets easily aggressive if we don't follow his schedule…”* *“… she becomes super angry if things don't go the way she wanted to go… zero tolerance if we force her to eat later than she prefers…”* *“… she has a very interesting routine. Every Monday she dresses up all blue and eats only pasta…”* *“… he can spend his entire time with puzzles, computer games, cycling and writing religious quotes…”*	Not available	Available data are not sufficient. Further efforts are warranted to encapsulate such deficits.

*Note*: **B**: Symptom can be reported as a behavioral problem by the caregiver. **L**: Symptom can be reported as a language problem by the caregiver. **M**: Symptom can be reported as a memory problem by the caregiver.

Although in the collected dataset, naming and word finding difficulties were frequently reported, available cognitive test scores revealed that almost all patients who had cognitive assessment had severe visual semantic deficits (pyramids and palm trees/pictures, 96%; famous faces naming, 100%; famous faces familiarity, 91%; famous faces semantic association, 100%, famous faces name familiarity,100%) but relatively less frequent naming problems in general naming tests (Boston Naming Test, 79%; Graded naming test, 87%; visual association task [VAT] naming, 50%). Additionally, the real‐life examples in the case notes and cognitive assessment results raised into question whether the reported symptoms were purely related to anomia or a retrieval problem. For instance, patients exhibited difficulties in naming their family members, but also showed lack of recognizing their family members both by their faces and voices. Of note, a loss of knowledge for landmarks, smells, flavors, sounds, and bodily sensations, inappropriate reactions to those stimuli, and changes in personal taste regarding food, colors, art, clothes, and other esthetic experiences were also reported, although no cognitive assessments focusing on recognition, naming, hedonic valuation, semantic association, and familiarity were available for these domains in our dataset (Tables 3 and 4).

In 67% of the cases, a memory deficit was rated as present and available cognitive test scores that assess only visual (Benson complex figure‐ delay, 57%, Visual Object Memory Test‐ delay, 82%, VAT‐ delay, 21%) and verbal memory (Rey Auditory Verbal Learning Test‐ recall, 57%, California verbal learning test recall, 62%) also confirmed memory deficits in this group, although the results varied across a wide range (Table 3). Unfortunately, the sample size for memory tests related to logical and social memory was insufficient to draw conclusive findings. In contrast to these findings, the empirical examples from the case notes suggested some discrepancies in episodic memory impairment. For example, patients were simply forgetting items listed in the previous section such as faces, landmarks, and so forth, but also had difficulty remembering some important events such as the 9/11 attacks. Some cases forgot the details of a birthday party that they attended last night but perfectly remembered their breakfast. Some of the cases also reported forgetting appointments, and recent events that might be considered as an episodic memory deficit (Table 4).

Last, the reported symptoms, case notes, and available cognitive/behavioral assessment tests helped to clarify the behavioral profile. Although mental rigidity and preoccupations were prominent and exhibited a wide spectrum, none of the participating centers utilized specific instruments to assess the nature and severity of these symptoms (Table 4). Disinhibition/socially inappropriate behavior was the most heterogeneous predefined category. The case notes revealed various domain impairments that could be categorized as “inappropriate behavior.” Examples included patients showing indifference toward spoiled food's distinct smell; making hyper‐focused, inefficient decisions; getting spammed by strangers; and being easily agitated due to mental rigidity (Table 4). Although an objective assessment for those behaviors was limited, the median Neuropsychiatric Inventory (NPI) score for disinhibition (frequency × severity) was 1 (min = 0, max = 12), reported in 53% of the patients. Furthermore, assessment using the Awareness of Social Inference Test (TASIT) sub‐domain for sarcasm revealed that all tested patients exhibited deficits in recognizing sarcasm and paralinguistic social cues, whereas sincere conversation comprehension was spared. Emotion recognition deficit, “caricatured” emotional reactions and misplaced empathy, and inappropriate/diminished emotional expressivity were prominently reported for “loss of empathy” (Table 4). Available cognitive test scores revealed that nearly all tested patients had an emotion recognition deficit (Comprehensive Affect Testing System [CATS] affect matching, 88%; TASIT Emotion Evaluation Test [EET], 92%). Informant‐based surveys showed a lack of empathic concern, perspective‐taking, and interpersonal coldness in 84%, 93%, and 89% of tested patients, respectively). Interestingly, only 33% of the tested patients exhibited impaired cognitive theory of mind (ToM) deficit, whereas all showed emotional ToM impairment. Social withdrawal and lack of enthusiasm for social activities were the main real‐life examples for “apathy” in the case notes (Table 4). In 126 patients with available NPI scores, apathy was observed in 88 individuals (69%), with a median frequency x severity rating of 2 (min = 0, max = 12).

## 4 DISCUSSION

This project is the first international collaboration and the largest reported cohort to elucidate the clinical profile of FTD associated with the predominant involvement of the RATL. In this first study of the IWG, we reported the retrospective chart reviews integrated with supportive cognitive assessment and real‐life examples reported by caregivers. The most common symptoms were mental rigidity and preoccupations (78%); disinhibition/socially inappropriate behavior (74%); recognition difficulties and altered reactions to taste, landmarks, bodily sensations, smell, and sound‐related stimuli (in total 74%); naming/word finding difficulties (70%); memory deficits (67%); apathy (65%); loss of empathy (65%); and face recognition deficits (60%). However, the integrated data unveiled that behavioral, language, and memory problems exhibited distinct traits in rtvFTD, warranting heightened precision. Additionally, while social cognition assessment was generally limited across the dataset, the available data revealed notable findings, including difficulties in emotion recognition, emotional perspective taking, and comprehending paralinguistic cues. Moreover, recognition deficits involving faces transcended mere visual perception, while recognition difficulties and altered reactions to sound, smell, taste, landmarks, and bodily sensations emerged as the most distinctive symptom category that awaits objective assessment in prospective cohorts. Among the most noteworthy findings was the utilization of varied terminologies for each symptom, underscoring gaps within our field. The underlying mechanisms behind these distinctive clinical presentations remain incompletely understood, and the lack of a standardized nomenclature is leading to clinical misdiagnoses and inaccurate registrations for clinical trials.

Historically, prosopagnosia has been considered a hallmark of rtvFTD. However, a large body of literature has suggested that the deficit in rtvFTD is different from posterior cortical areas related to face perception deficit, but associated with a multi‐modal deficit in knowledge about people encompassing their voices, biographical information, and their names as well as faces which has been termed as “person‐specific knowledge.”^9^ This holds significance in distinguishing these cases from other dementia subtypes, necessitating a revision of our symptom descriptions and patient assessment protocols.

Beyond person‐specific knowledge, our case notes revealed that rtvFTD patients also encounter challenges in recognizing landmarks, smell, taste, sound, and bodily sensations, and exhibited inappropriate reactions to those stimuli, despite the fact that objective assessments of those domains were not available in our dataset. Previous publications have shown the semantic deficits for landmarks,^3^, ^4^ sound/audio,^17^, ^18^ smell/odor,^19^ taste/flavor,^20^ bodily sensations/interoceptive/somatic signals,^15^, ^21^ pain, and temperature^22^, ^23^ in small samples. However, it is still debatable whether chemosensory alterations (taste, smell) are due to a pure semantic deficit or a broader deficit of hedonic valuation.^14^ Noteworthy, many of these symptoms were reported within the categories of “naming/word finding difficulties,” “disinhibition/socially inappropriate behavior,” or “memory deficits,” underscoring the imperative of adopting a unified and meaningful nomenclature. Another important nuance in this category is altered responses of rtvFTD patients to bodily sensations. This symptom is ubiquitous in the rtvFTD literature; mostly termed as “somatization,” “hypochondria,” or “alexisomia,”^7^, ^15^ although now the lack of semantic knowledge for interoceptive stimuli has been suggested as the underlying mechanism.^15^ Nevertheless, all those hypotheses are yet to be tested in larger rtvFTD cohorts to elucidate the neural mechanisms and determine whether they are early characteristic symptoms of the syndrome.

All symptoms listed above may easily be considered as behavioral problems, like those occurring in bvFTD, although in most instances they could potentially arise from the loss of semantic knowledge. Additionally, even though diet changes occur in both subtypes, at a closer look, narrowed food preferences are prevalent in rtvFTD instead of hyperorality or sweet tooth which are common in bvFTD literature.^24^ Furthermore, rather than simple repetitive (i.e., pacing, tapping, and picking) or stereotypic motor behavior, in rtvFTD, rigid preoccupations leading to complex repetitive behaviors are prominent.^7^, ^9^ which vary from emergence of artistic skills to clock watching.^1^, ^6^, ^7^, ^8^, ^9^ Next to those specific interests, alterations in personal preferences (not only food, but also colors, clothes, and aesthetic taste), mental rigidity, and narrowed thought processes that may also cause altered decision‐making were observed in the international data. The understanding of these behaviors is hampered by a limited body of research. Real‐life examples provided in our study illustrate that the nature of these symptoms differs from primary obsessive‐compulsive disorder, which is typically more anxiety‐ or self‐criticism‐oriented, occurring with complete insight.^25^ Instead, they seem to align more with a set of overvalued ideas, rigid thinking, inflexibility, and rumination. Despite the high prevalence of these symptoms (78%), the underlying cognitive processes and functional anatomy are not well‐resolved. Future work will enlighten the equivocality of those acquired new features. Other barriers in disentangling the behavioral profile in rtvFTD are limited semiology described by broad terms like disinhibition, apathy, and loss of empathy and the assessment heavily relying on self/caregiver‐reported symptoms and questionnaires. A recent conceptual framework has offered an anatomical model for the umbrella term “disinhibition” suggesting that ATL‐related disinhibition is associated with loss of knowledge of social norms and expectations rather than a control problem.^26^ Echoing this argument, a large body of literature supports that conceptual knowledge of social constructs and socially relevant cues are represented in the ATLs.^13^, ^27^, ^28^

Besides, the involvement of RATL in empathy processing is widely documented.^28^ However, clinical descriptions have often been limited to a broad term “loss of empathy” lacking the granularity concerning specific cognitive abilities and underlying mechanisms. While the role of RATL in emotion recognition, empathic concern, and perspective has been studied, recent research showed lower scores in emotional ToM in rtvFTD, in contrast to patients with frontal bvFTD who perform worse in cognitive ToM tasks,^9^ underscoring the importance of objective assessments in the differential diagnosis of these conditions. This distinction could have significant implications for the examination of other behavioral symptoms such as apathy, specifically in the disentanglement of its cognitive and emotional dimensions. Our findings indicate that rtvFTD patients tend to lose enthusiasm for social interactions while remaining highly motivated in solitary preoccupations. This contrasts with classical cognitive apathy, urging an exploration of RATL's role in motivation.

The occurrence of amnestic presentations in rtvFTD is another controversial topic. To date, episodic, semantic, and autobiographical memory deficits have been documented with discrepant frequencies by several groups.^4^, ^7^, ^8^, ^29^, ^30^ In our study, although chart reviews showed that 67% of patients had memory problems, the results varied between 21% and 87% across eight different memory tests. Furthermore, the nature of the real‐life examples necessitated a deeper investigation of this symptom. One lingering question remains: how does amnesia persist in rtvFTD, even in amyloid‐negative cohorts?^7^ Could it involve the role of semantics in episodic memory beyond semantic memory,^30^ or the possible presence of hippocampal sclerosis in such cases?^31^ Given the upcoming therapies for AD and FTD, elucidating the nature of the memory deficit in rtvFTD is pivotal to avoid misdiagnosis not only with AD but also with other FTLD subtypes presenting with amnesia.

Besides those core symptoms, affective dysregulation, anxiety/panic, delusions/hallucinations, motor problems, word comprehension, and object recognition deficits were also observed with lower frequencies in line with previous publications.^7^, ^8^ Although, previous literature suggested depression as a distinctive symptom,^7^, ^8^ our joint data also showed cases with mania and fluctuating mood. Last, visuospatial problems were the least rated symptoms, and cognitive test scores highlighted spared visuospatial functions that might assist diagnosis in daily practice.

Despite the novelty and impact of the project, it is important to acknowledge certain nuances and limitations. Cases without genetic and/or pathological confirmation were not excluded, as the majority of individuals were sporadic. Additionally, the use of visual rating scores, although conducted by experienced experts, may be considered relatively subjective, and employing measures such as CDR FTLD or CDR box scores instead of global CDR could enhance the identification of very early‐stage patients. Furthermore, the absence of a direct comparison between rtvFTD and its differential diagnoses, such as bvFTD and svPPA, limits the interpretation of the observed symptoms' sensitivity and specificity. Last, due to the nature of a retrospective design, the main limitations were unnoticed symptoms in historical case notes and a lack of systematic objective assessments for many RATL‐related domains. Nonetheless, a key strength of this study lies in its provision of previously absent measurements and the harmonization of outcomes derived from real‐life concerns expressed by caregivers, clinicians' interpretations, and cognitive assessments. These elements challenge our routine clinical assessments and lay a robust foundation for the IWG's forthcoming endeavors.

As described in this study, the nature of many RATL symptoms remains poorly understood and vaguely characterized. The anatomical underpinnings, including the contribution of co‐existing atrophies and altered network dynamics following focal neurodegeneration, are still awaiting detailed investigation. Our ultimate goals are reaching a consensus on nomenclature and diagnostic criteria, defining the core symptoms of the syndrome, and employing precise terminologies that accurately reflect neural mechanisms. The overarching aim is to facilitate the recognition of FTD associated with RATL atrophy and differentiate the syndrome from bvFTD, svPPA, AD, and psychiatric disorders.

## CONFLICT OF INTEREST STATEMENT

The funders of the study had no role in study design, data collection, data analysis, data interpretation, or writing of the report. Oskar Hansson has acquired research support (for the institution) from ADx, AVID Radiopharmaceuticals, Biogen, Eli Lilly, Eisai, Fujirebio, GE Healthcare, Pfizer, and Roche. In the past 2 years, he has received consultancy/speaker fees from AC Immune, Amylyx, Alzpath, BioArctic, Biogen, Cerveau, Eisai, Eli Lilly, Fujirebio, Merck, Novartis, Novo Nordisk, Roche, Sanofi, and Siemens. Other authors declare no competing interest. Author disclosures are available in the supporting information.

## CONSENT STATEMENT

All participating centers obtained ethical approval from their local committees, and all human subjects provided informed consent.

## Supporting information

Supporting Information

Supporting Information

## COLLABORATORS: Investigators of the IWG

Alan Lerner^31^, Alexander F Santillo^12^, Alexandre Morin^32^, Ana Manera^5^, Arabella Bouzigues^33^, Bedia Samanci^8^, Bradley Boeve^28^, Bruce Miller^2^, Carmela Tartaglia^34^, Caroline Dallaire Theroux^18^, Christopher Kobylecki^11^, Daniela Galimberti^23,24^, David Foxe^35^, David Perry^2^, Diana Matallana^36^, Elisabet Englund^12^, Emily Rogalski^37^, Emma Devenney^35^, Francesco Di Lorenzo^38^, Frederik Barkhof^22,23^, Fiona Kumfor^35^, Florence Pasquier^3^, Gail Robinson^39^, Giorgio Fumagalli^6,7^, Giuseppe Piga^33^, Gregory Kuchcinski^3^, Hakan Gurvit^8^, Halle Quang^35^, Harro Seelaar^40^, Howie Rosen^2^, Hulya Ulugut^1,2^, Ignacio Illan Gala^9^, James Rowe^41^, Jan Van den Stock^16^, Janine Diehl Schmid^15^, Jason Warren^27^, Jennifer C. Thompson^10^, Jennifer Whitwell^29^, Jessica Hazelton^35^, Jonathan Rohrer^27^, Julie Fields^28^, Julie Snowden^10^, Julien Lagarde^42^, Jwala Narayanan^43^, Katherine Rankin^2^, Keith Josephs^28^, Kristina Horne^35^, Kyan Younes^2,4^, Leonardo Cruz de Souza^20^, Leonel Takada^21^, Lina Riedl^15^, Lize Jiskoot^40^, Lucy Russell^27^, Maria Landqvist Waldo^14^, Maria Luisa Gorno Tempini^2,30^, Marsel Mesulam^37^, Masud Husain^44^, Mathieu Vandenbulcke^16^, Matthew Jones^10^, Matthew Rouse^41^, Maud Tastevin^18^, Maxime Bertoux^3^, Maxime Montembeault^2,5^, Mira Didic^45,46^, Murat Emre^8^, Muireann Irish^35^, Nick C. Fox^27^, Olivier Piguet^35^, Oskar Hansson^12^, Philip Scheltens^1^, Paulo Caramelli^20^, Peter Pressman^19^, Raffaella Migliaccio^33^, Ratnavallie Ellajosyula^43^, Rik Ossenkoppele^25^, Rik Vandenberghe^47^, Robert Laforce^18^, Samantha Loi^48^, Sandra Weintraub^37^, Shalom Henderson^41^, Sian Thompson^41^, Siddharth Ramanan^41^, Simon Ducharme^5^, Thibaud Lebouvier^3^, Toji Miyagawa^28^, Virginia Sturm^2^, Welmoed Krudop^1^, Wiesje van der Flier^1^, Yolande Pijnenburg^1^

^1^Alzheimer Center Amsterdam, Department of Neurology, Amsterdam Neuroscience, Vrije Universiteit Amsterdam, Amsterdam UMC, Amsterdam, The Netherlands, ^2^Memory and Aging Center, Department of Neurology, UCSF Weill Institute for Neurosciences, University of California, San Francisco, USA, ^3^The University of Lille, Inserm, Lille Neurosciences & Cognition Institute, Lille, France, ^4^Stanford Neuroscience Health Center, Department of Neurology, Stanford University, Palo Alto, USA, ^5^Department of Psychiatry, McGill University Health Centre, McGill University, Montreal, Canada, ^6^Department of Neurology, University of Milan, Milan, Italy, ^7^Università degli Studi di Trento | UNITN · CIMEC—Center for Mind/Brain Sciences, ^8^Department of Neurology, Istanbul University, Istanbul, Turkey, ^9^Department of Neurology, Hospital de la Santa Creu I Sant Pau, Barcelona, Spain, ^10^Cerebral Function Unit, Greater Manchester Neuroscience Centre, Salford Royal NHS Foundation Trust, Salford, UK; Division of Neuroscience and Experimental Psychology, Faculty of Biology, Medicine and Health, University of Manchester, Manchester, UK, ^11^Department of Neurology, Manchester Centre for Clinical Neurosciences NHS Foundation Trust, Salford, UK; Division of Neuroscience, University of Manchester, Manchester, UK^12^Department of Clinical Sciences, Clinical Memory Research Unit, Faculty of Medicine, Lund University, Lund/Malmö, Sweden, ^13^Division of Pathology,Department of Clinical Sciences,Lund University,Lund,SE‐221 85,Sweden, ^14^Division of Clinical Sciences Helsingborg, Department of Clinical Sciences Lund, Lund University, Lund, Sweden, ^15^School of Medicine, Department of Psychiatry and Psychotherapy, Technical University of Munich, Munich, Germany, ^16^Laboratory for Translational Neuropsychiatry, Department of Neurosciences, KU Leuven, Leuven, Belgium, ^17^Department of Neurology, University Hospital Leuven, Leuven, Belgium, ^18^Clinique Interdisciplinaire de Mémoire (CIME), Laval University, Quebec, Canada, ^19^University of Colorado, Anschutz Medical Campus, Behavioral Neurology Section, Department of Neurology, USA, ^20^Behavioral and Cognitive Neurology Unit, Department of Internal Medicine, Faculdade de Medicina, Universidade Federal de Minas Gerais, Belo Horizonte, Brazil, ^21^Cognitive and Behavioral Unit, Hospital das Clinicas, Department of Neurology, University of São Paulo Medical School, São Paulo, Brazil ^22^ Kbo‐Inn‐Salzach‐Klinikum, Clinical Center for Psychiatry, Psychotherapy, Psychosomatic Medicine, Geriatrics and Neurology, Wasserburg/Inn, Germany ^23^Department of Biomedical, Surgical and Dental Sciences, University of Milan, Milan, Italy, ^24^Fondazione IRCCS Ca’ Granda, Ospedale Maggiore Policlinico, Milan, Italy ^25^Alzheimer Center Amsterdam, Department of Radiology, Amsterdam Neuroscience, Vrije Universiteit Amsterdam, Amsterdam UMC, Amsterdam, The Netherlands, ^26^UCL Institutes of Neurology and Healthcare Engineering, University College London, ^27^Dementia Research Centre, Institute of Neurology, Queen Square, London, UK, ^28^Department of Neurology, Mayo Clinic, Rochester, Minnesota, USA, ^29^Department of Radiology, Mayo Clinic, Rochester, Minnesota, USA, ^30^Dyslexia Center, University of California, San Francisco, USA ^31^Department of Neurology, Case Western Reserve University, Cleveland, OH,USA, ^32^Department of Neurology Rouen University Hospital, France, ^33^ICM Paris Brain Institute, Paris France, ^34^Tanz Centre for Research in Neurodegenerative Diseases, University of Toronto, Toronto, Canada, ^35^Brain and Mind Centre, University of Sydney, Sydney, Australia, ^36^Department of Neurology, Javeriana University, Bogotá, Colombia, ^37^Mesulam Center for Cognitive Neurology and Alzheimer's Disease Northwestern University Feinberg School of Medicine, Chicago, 60611 IL, ^38^Non‐invasive Brain Stimulation Unit, IRCCS Fondazione Santa Lucia, Rome, Italy, ^39^Queensland Brain Institute & School of Psychology, The University of Queensland, Brisbane, Australia, ^40^Department of Neurology, Erasmus MC—University Medical Centre Rotterdam, Rotterdam, The Netherlands, ^41^Department of Clinical Neurosciences and Cambridge University Hospitals NHS Trust and Medical Research Council Cognition and Brain Sciences Unit, University of Cambridge, Cambridge, UK ^42^Department of Neurology of Memory and Language, GHU Paris Psychiatrie & Neurosciences, Hôpital Sainte Anne, Paris, France ^43^Department of Neurology, Manipal Hospital India, Bengaluru, Karnataka, India, ^44^Oxford University Hospitals NHS Foundation Trust, Oxford, UK, ^45^AP‐HM, Service de Neurologie et Neuropsychologie, Pole de neurosciences cliniques, Timone, Marseille, France ^46^Aix‐Marseille Univ, Institut des Neurosciences de Systèmes, Inserm U1106, Marseille, France, ^47^Neuropsychiatry Centre, Royal Melbourne Hospital and Department of Psychiatry, University of Melbourne, Australia.
