# Supplementary material for: Clinical recognition of frontotemporal dementia with right anterior temporal predominance: A multicenter retrospective cohort study
Source: Alzheimers Dement. 2024 Jul 10;20(8):5647–61. doi: 10.1002/alz.14076 (PMC11350044; doi:10.1002/alz.14076)

Clinical Recognition of Frontotemporal Dementia with Right Anterior Temporal Predominance; Progress Report of the International Working Group

SUPPLEMENTARY MATERIALS

Contents

1. Member list of the international working group

Page 2

2. Roadmap of the international working group

Page 5

3. Establishment of the international working group

Page 5

4. Consensus approach

Page 5

5. Patient selection

Page 6

6. Data collection, harmonization of the data, and approach on missing data

Page 6

7. Analysis

Page 7

8. Guidebook for data collection

Page 9

9. Data sharing procedure

Page 16

10. Available cognitive tests in the collected dataset

Page 17

11. Comparison of the centers

Page 18

Member list of the international working group (Alphabetical order)

1. Alan Lerner, Case Western Reserve University
2. Alexander F Santillo, Lund University
3. Alexandre Morin, Rouen University Hospital
4. Ana Manera, McGill University
5. Arabella Bouzigues, ICM Paris Brain Institute
6. Bedia Samanci, Istanbul University
7. Bradley Boeve, Mayo Clinic Rochester
8. Bruce Miller, UCSF Memory and Aging Center
9. Carmela Tartaglia, University of Toronto
10. Caroline Dallaire Theroux, University of Laval
11. Christopher Kobylecki, Manchester Cerebral Function Unit
12. Daniela Galimberti, University of Milan
13. David Foxe, University of Sydney
14. David Perry, UCSF Memory and Aging Center
15. Diana Matallana, Javeriana University
16. Elisabet Englund, Lund University
17. Emily Rogalski, Northwestern University
18. Emma Devenney, University of Sydney
19. Francesco Di Lorenzo, Foundation Santa Lucia
20. Frederik Barkhof, Alzheimer Center Amsterdam
21. Fiona Kumfor, University of Sydney
22. Florence Pasquier, University of Lille
23. Gail Robinson, The University of Queensland
24. Giorgio Fumagalli, University of Milan
25. Giuseppe Piga, ICM Paris Brain Institute
26. Gregory Kuchcinski, University of Lille
27. Hakan Gurvit, Istanbul University
28. Halle Quang, University of Sydney
29. Harro Seelaar, Erasmus University
30. Howie Rosen, UCSF Memory and Aging Center

31. Hulya Ulugut, UCSF Memory and Aging Center
32. Ignacio Illan Gala, Hospital de la Santa Creu I Sant Pau
33. James Rowe, Cambridge University
34. Jan Van den Stock, Leuven University
35. Janine Diehl Schmid, Technical University of Munich
36. Jason Warren, Dementia Research Centre London
37. Jennifer Thompson, Manchester Cerebral Function Unit
38. Jennifer Whitwell, Mayo Clinic Rochester
39. Jessica Hazelton, University of Sydney
40. Jonathan Rohrer, Dementia Research Centre London
41. Julie Fields, Mayo Clinic Rochester
42. Julie Snowden, Manchester Cerebral Function Unit
43. Julien Lagarde, Hospital Sainte Anne
44. Jwala Narayanan, Manipal Hospital India
45. Katherine Rankin, UCSF Memory and Aging Center
46. Keith Josephs, Mayo Clinic Rochester
47. Kristina Horne, University of Sydney
48. Kyan Younes, Stanford University
49. Leonardo Cruz de Souza, Universidade Federal de Minas Gerais
50. Leonel T. Takada, University of Sao Paulo
51. Lina Riedl, Technical University of Munich
52. Lize Jiskoot, Erasmus University
53. Lucy Russell, Dementia Research Centre London
54. Maria Landqvist Waldo, Lund University
55. Maria Luisa Gorno Tempini, UCSF Memory and Aging Center
56. Marsel Mesulam, Northwestern University
57. Masud Husain, Oxford University
58. Mathieu Vandenbulcke, Leuven University
59. Matthew Jones, Manchester Cerebral Function Unit
60. Matthew Rouse, Cambridge University
61. Maud Tastevin, University of Laval

62. Maxime Bertoux, University of Lille
63. Maxime Montembeault, McGill University
64. Mira Didic, Aix-Marseille University
65. Murat Emre, Istanbul University
66. Muireann Irish, University Sydney
67. Nick Fox, Dementia Research Centre London
68. Olivier Piguet, University of Sydney
69. Oskar Hansson, Lund University
70. Philip Scheltens, Alzheimer Center Amsterdam
71. Paulo Caramelli, Universidade Federal de Minas Gerais
72. Peter Pressman, University of Colorado
73. Raffaella Migliaccio, Pitie-Salpetriere Hospital, INSERM
74. Ratnavallie Ellajosyula, Manipal Hospital India
75. Rik Ossenkoppele, Alzheimer Center Amsterdam
76. Rik Vandenberghe, Leuven University
77. Robert Laforce, University of Laval
78. Samantha Loi, University of Melbourne
79. Sandra Weintraub, Northwestern University
80. Shalom Henderson, Cambridge University
81. Sian Thompson, Oxford University
82. Siddharth Ramanan, Cambridge University
83. Simon Ducharme, McGill University
84. Thibaud Lebouvier, University of Lille
85. Toji Miyagawa, Mayo Clinic Rochester
86. Virginia Sturm, UCSF Memory and Aging Center
87. Welmoed Krudop, Alzheimer Center Amsterdam
88. Wiesje van der Flier, Alzheimer Center Amsterdam
89. Yolande Pijnenburg, Alzheimer Center Amsterdam

Roadmap of the International Working Group (IWG)

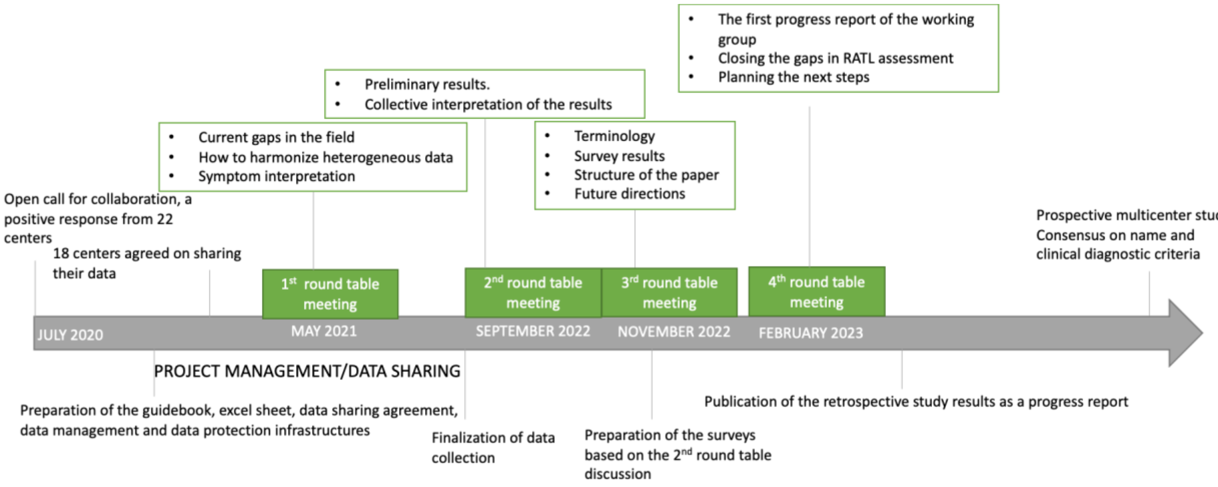

Establishment of the IWG: HU and YP reviewed the international literature to identify the authors / centers that might have an interest in rtvFTD, based on their scientific reports and /or clinical cohort characteristics. Partners were invited via email and/or zoom meetings to collaborate in the project. Initial invitations were extended to multiple centers, and subsequent Zoom meetings were held to outline the objectives of the IWG and the specific project. This resulted in a positive reaction for collaboration from 18 centers including the United States, United Kingdom, Italy, France, Belgium, Germany, Spain, Sweden, Canada, Turkey, Brazil, and the Netherlands. The invitation remained open to allow additional centers to join subsequent round table meetings, which aimed to achieve consensus on various aspects including aims, methodology, study designs, generation of the symptom checklist for data collection, data analyses, interpretation of the collected data, terminology, recommendations, and future directions. Over time new collaborators have joined the collaboration that contributed to the round table discussions although they couldn't provide patient data for the retrospective study. Currently the IWG contains 30 centers across the world with 90 early career and senior FTD researchers.

Consensus approach: A series of round table meetings was organized to reach consensus on aims, methodology, generation of the symptom checklist, study designs and future directions. The nominal group process technique which combines qualitative and quantitative data and encourages and enhances the participation of group members was used to deliver rapid and reliable results to have a consensus and generate ideas/solutions to be used in the project

(*GALLAGHER M, HARES T, SPENCER J, BRADSHAW C, WEBB I. The Nominal Group Technique: A Research Tool for General Practice? Fam Pract. 1993 Mar 1;10(1):76–81*). All meetings were recorded and shared on safe platforms (SURFDrive). Generation of the symptom checklist, symptom definitions, interpretations, terminology, and how to abstract data from case notes were elucidated and a guidebook, open discussion chat (SURFDrive), and a dataset template were prepared based on the discussions. Following data collection, preliminary results such as sample characteristics, frequencies of the symptoms and cognitive test results were documented, and surveys were prepared to systematically collect the opinions of the collaborators. All results were shared and discussed in the round table meetings to (1) interpret and categorize the listed symptoms and (2) establish consensus on most frequent and distinctive symptoms and recommended terminology.

Patient selection: Among subjects registered with a clinical diagnosis of FTD and/or PPA those who demonstrated predominant RATL atrophy on the initial neuroimaging were selected. For identifying the RATL predominant atrophy, all FTD patients whose initial neuroimaging available were included. A guidebook was prepared for visual rating to score the atrophy. Visual atrophy rating was conducted by either an experienced radiologist or a dementia expert in each center by using our guidelines (see below). If the atrophy on the RATL was higher than the non-RATL areas (left temporal or frontal), this patient was considered as an rtvFTD case. Patients with frontal/left temporal atrophy ≥ 3 (max score=4) (even if they had predominant right temporal atrophy) were excluded to minimize gross effects of general neurodegeneration.

Data collection, harmonization of the data, and approach on missing data: The data were collected from the case notes retrospectively (End date: July 2022). A dataset template was prepared based on the published RATL degeneration/temporal variant FTD literature and round table discussions to fill in the pseudonymised data which comprises (1) demographic, (2) clinical data (including symptoms, family history, dementia severity, and cognitive tests and neuropsychiatric inventory (NPI) scores), (3) atrophy rating scores for each anatomical area, (4) biomarkers (CSF amyloid, tau, amyloid PET, neurofilament light chain (NfL) or other potential biomarkers for FTD-if available), (5) genetic mutations, (6) pathological information. A broad range of clinical symptoms (cognitive, behavioral, language, psychiatric, other) was coded as present, absent or not available at the initial and follow-up visits. If it is present, the collectors were asked to describe the symptom by copying the real-life examples from the case notes.

Frequencies were calculated based on the available data, excluding any missing data. No imputation technique was used to deal with the missing data since one of the goals was identifying the gaps as well. The number of available data for each symptom is displayed alongside the corresponding percentage in the results. Additionally, there was an “other” column for each section where the collectors were able to add more data/information that was not on the predefined list. All data in this section were checked by HU and YP to avoid duplications. If the details were already mentioned in other columns, they were kept as real-life examples. The rest was analyzed and similar symptom descriptions (ie: for landmarks: “topographagnosia”, “problems with naming landmarks”, “cannot recognize buildings”, for taste: “gustatory agnosia”, “cannot differentiate the taste”, “flavor recognition problem” etc.,) were clustered to be discussed in the round table meetings. To provide safe, open access and transparent data, SURFdrive and CASTOR softwares were used and separate data sharing agreements were signed with each center to protect the rights of the research participants and data suppliers.

Analysis: Variables were reported as means and standard deviations or proportions when appropriate. The frequency of individual symptoms was reported as proportions. All cases had symptom check list charts generated by the IWG and nearly half of the sample had details of the case notes (real life examples) for each symptom (n=134). Symptoms were listed based on their frequencies at the initial visit and each reported symptom was associated with real life examples and available cognitive tests. The list of the tests can be found below. ***Harmonization of the cognitive test scores:*** To harmonize the heterogeneous cognitive test scores, we used the following methodology. Based on the numbers and the relevance of the tests based on the published literature, a selection was made for each cognitive domain. Similar test scores of widely used tests were combined. If centers shared different versions of the well-known tests, the scores were adapted based on the published literature. Uncommon scoring systems were excluded even if a well-known test was used (e.g.: seconds instead of longest span in the digit span tests). Z-scores were calculated with the formula: (patient’s score)-mean (norm)/SD(norm) and a z score lower than -1.65 was considered as impaired for each individual. Z scores couldn’t be calculated for some tests that had small sample sizes and had no healthy control data. For interpretation of those tests, cut off scores were used. In the table, percentages of the patients whose z scores are lower than -1.65 and/or cut off value are displayed.

Guidebook for data collection

The excel file contains 4 pages. Page 1 is designed for initial features. Page 2 and 3 are for follow-up visits. Page 4 is for abbreviations and coding system. If your patient does not have a follow-up visit, please type not available (n.a.) into the follow-up pages. If your patient has more than 2 follow up visits please copy the template and create a new follow-up page. We prepared 2 sample cases as an example for you.

The excel page comprises 8 headings including demographical features (1), clinical symptoms (2), family history (3), dementia severity and cognitive assessment (4), neuropsychological assessment (5), neuroimaging (6), biomarker (7), genetic and pathological confirmation (8).

1. **DEMOGRAPHICS**

This heading comprises 5 subheadings

1. CASE: Data must be anonymized. Please do not share personal ID data. The case ID will be the combination of your city and the case number (ie: Montreal12)
2. Age: years
3. Gender: female (code:0), male (code:1)
4. Symptom duration: from symptom onset to initial visit. Years

NOTE: In the follow pages you will see follow-up duration. Please be sure that you typed the duration from initial visit to follow-up visit. Months (Not years)

5. Handedness: right, left, ambidexter. (If your patient is not right handed please mention the hemispheric dominance)

2. **CLINICAL SYMPTOMS**

This heading comprises 5 subheadings. If the mentioned symptom is present in the case notes, please type 1, if it is absent type 0. If it is not mentioned in the case notes, please type "n.a.". This part is the limitation of our retrospective study design. However, we expect that characteristic dementia symptoms are systematically asked in the tertiary care dementia centers. Additionally, if your patient has additional symptoms please write those symptoms into the "other" column.

3. **FAMILY HISTORY**

Here you will see 4 subheadings. For dementia, please score the family history based on modified Goldman score (Please see below, *Rohrer et al., 2009. The heritability and genetics of frontotemporal lobar degeneration*)

<u>*MODIFIED GOLDMAN SCALE*</u>

1 is an autosomal dominant family history of FTLD, MND, CBS, or PSP, defined as the presence of at least 3 affected people in 2 generations with 1 person being a first-degree relative of the other 2,

2 is familial aggregation of 3 of more family members with dementia but not meeting criteria for 1,

3 is 1 other affected family member with dementia (modified to give a score of 3 only if there is a history of young-onset dementia within the family, i.e., less than 65, and 3.5 if onset above 65),

4 is no or unknown family history.

If the family history is specifically asked for and if it is negative, please type “0”. If the patient has a positive family history for psychiatric disorders, amyotrophic lateral sclerosis (ALS) or other neurodegenerative disorders please write in detail (see sample cases).

4. **DEMENTIA SEVERITY AND COGNITIVE ASSESSMENT**

In this section, we used the routine tests and scales for assessing the dementia severity and the cognitive impairment in the Alzheimer Centrum Amsterdam (for the details of the tests and daily patient assessment please see; *Van der Flier M. and Scheltens P., 2018. Amsterdam Dementia Cohort: Performing Research to Optimize Care*). If your center uses the same tests please fill in the excel file, otherwise please prepare a separate cognitive assessment table. Of note, for statistical analysis, after collecting the data, we’ll need either z scores or comparison with healthy controls.

5. **NEUROPSYCHOLOGICAL ASSESSMENT**

Similar to item 4. However, for neuropsychiatric inventory (NPI), please use multiplication of the frequency and severity scores. For instance; depression frequency score: 2, depression severity score: 1, frequency X severity= 2.

6. **NEUROIMAGING**

Right anterior temporal lobe (RATL) predominant impairment is the inclusion criterion of our study. Therefore, all cases have to be examined by at least one structural neuroimaging technic. Since the specificity of PET imaging is relatively low, we considered to include only MRI or CT.

We will use the visual assessment method to identify the RATL atrophy. For the mesial temporal atrophy (MTA) scoring please see below (*Scheltens et al., 1992 Atrophy of medial temporal lobes on MRI in "probable" Alzheimer's disease and normal ageing: diagnostic value and neuropsychological correlates*). For the frontotemporal atrophy scoring please see below (*Kipps et al., 2007 Clinical Significance of Lobar Atrophy in Frontotemporal Dementia: Application of an MRI Visual Rating Scale*). Additionally you will see a protocol for anterior temporal atrophy rating (Credit: Giorgio Fumagalli and also *Harper et al., 2015 Using visual rating to diagnose dementia: a critical evaluation of MRI atrophy scales*) The most important issue is that the frontal or the left temporal atrophy scores must be lower (at least 1 grade) than the RATL atrophy score. Patients with frontal/left temporal atrophy ≥ 3 (max score=4) (even if they had predominant RATL atrophy) will be excluded to minimize gross effects of general neurodegeneration.

1. **Anterior Temporal Rating Protocol**

1.a. SLICE SELECTION

Select the first slice where the connection between the frontal and temporal lobes is NOT visible: rate temporal pole

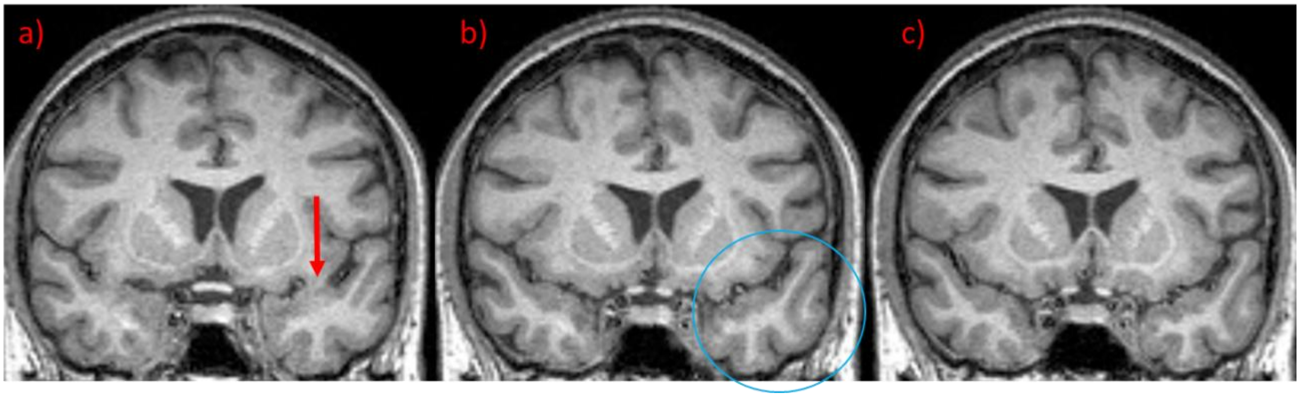

a) Pre-rating slice: connection between the frontal and temporal lobes is still visible

b) Correct slice: NO visible connection between the frontal and temporal lobes

c) Post-rating slice

1.b. RATING GUIDE

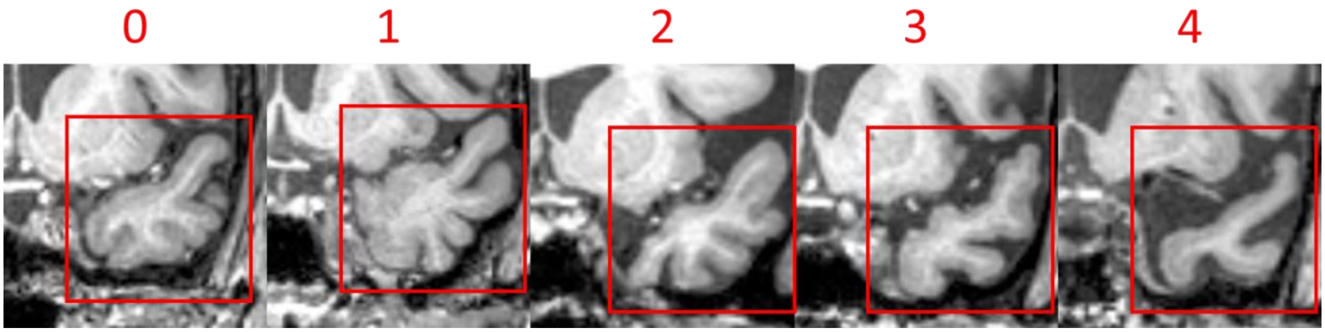

0: Normal appearances (closed sulci)

1: Small sulcal slit of anterior temporal sulci

2: Temporal sulci definitely widened

3: Triangular shape of gyri

4: Severe atrophy of Temporal pole (convex sulci)

2. **Scheltens MTA Scale**

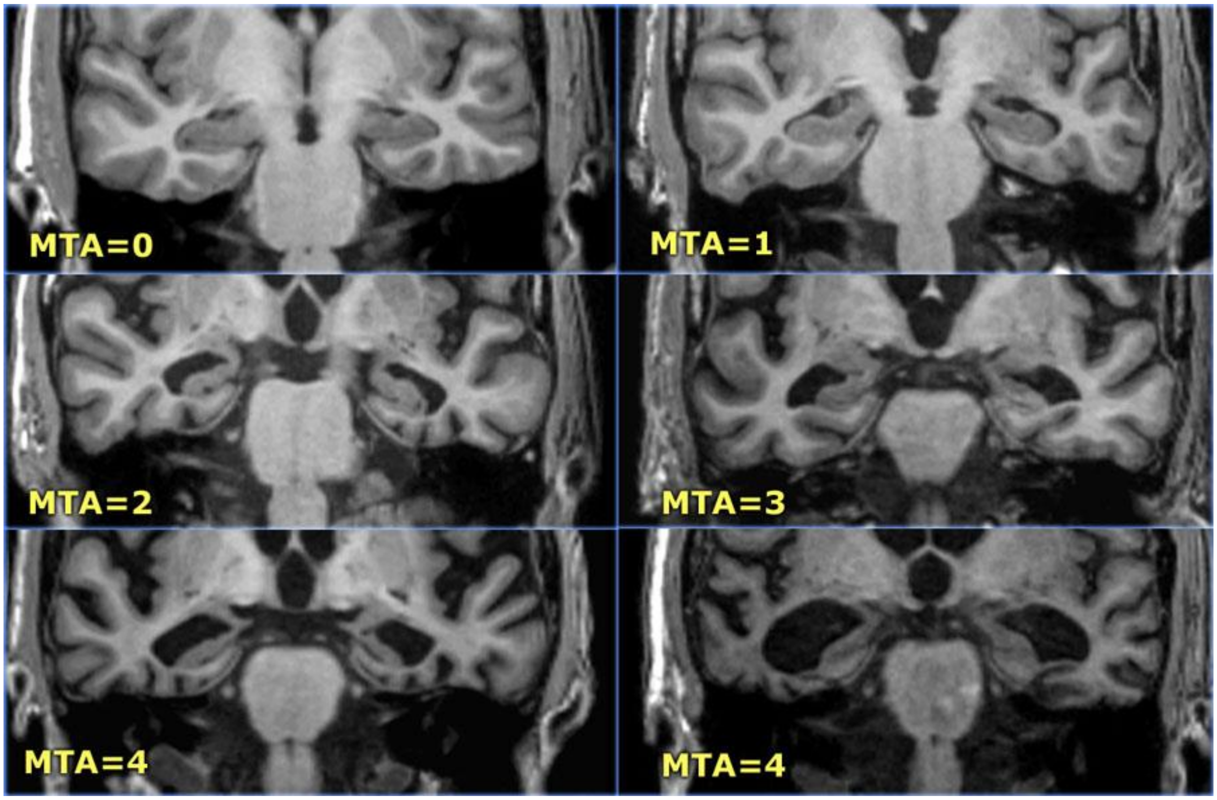

3. **Kipps Frontotemporal Scale**

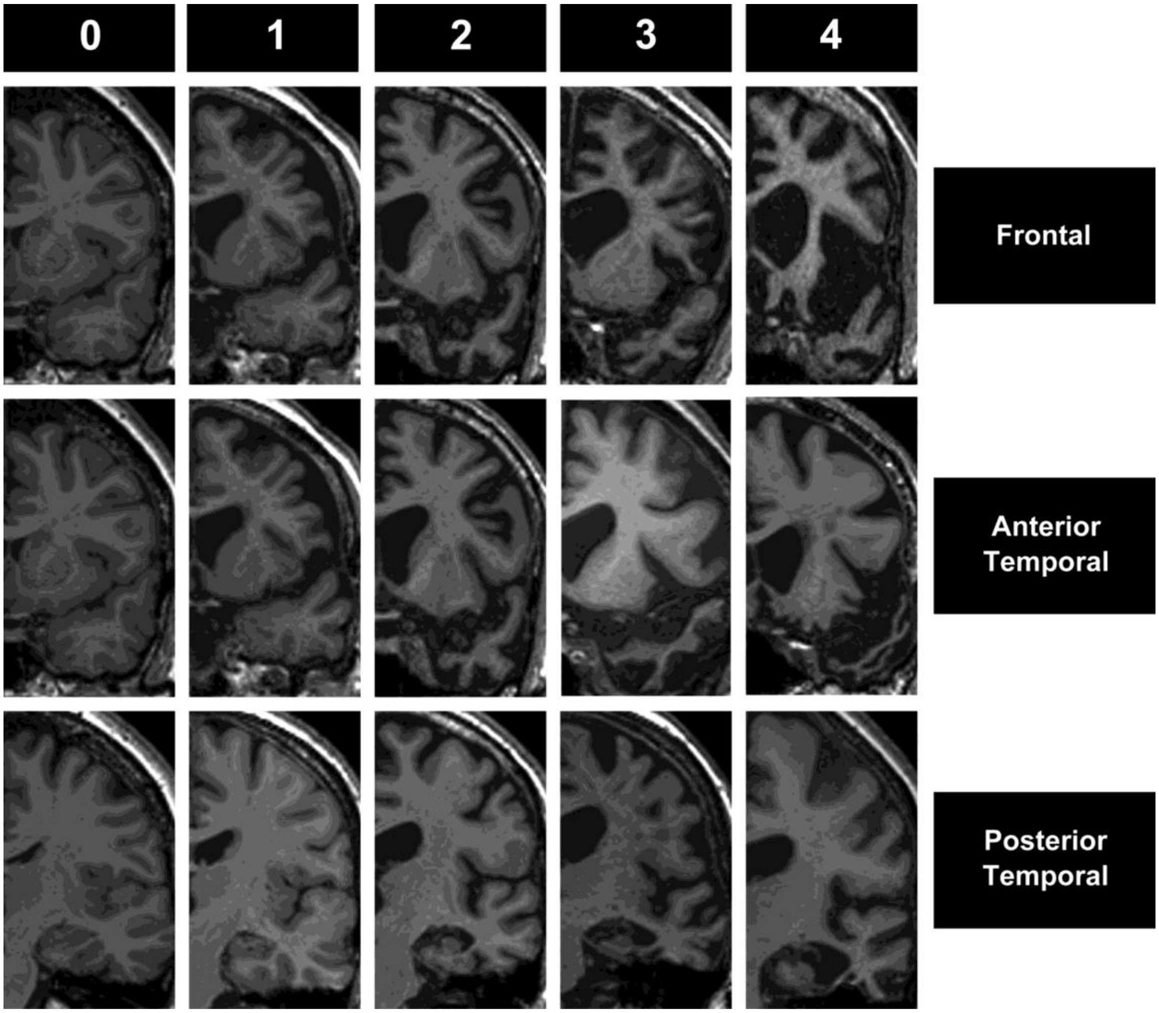

Image is from *Harper et al., 2015 Using visual rating to diagnose dementia: a critical evaluation of MRI atrophy scales*

7. **BIOMARKER**

If the amyloid status of your patient is not available, please type "n.a." If the CSF or amyloid PET result of your patient is suggestive for Alzheimer's Disease, please type "1", if not in other words amyloid negative, please type "0". If you have the neurofilament data such as neurofilament light chain (NfL) or other potential biomarkers please write them into the respective column.

8. **GENETIC AND PATHOLOGICAL CONFIRMATION**

In this part, we will use the same coding system. Positive: 1, negative: 2 and Not available: n.a.
If the patient has a genetic mutation please write the locus in the file. (ie: MAPT; Arg406Trp) for C9orf72, please write the number of repeat.

For follow visits, follow-up duration time and clinical data will be sufficient.

Data Sharing Procedure

In this retrospective multicenter project, Alzheimer Center Amsterdam (ACA), will be responsible to control the entire data management process along the research lifecycle; from study preparation, data acquisition, data processing and statistical analysis, writing and publishing, to archiving and open data, in a transparent and traceable way. Our data management plan is prepared under the supervision of our professional research support team (Amsterdam UMC, Locatie VUmc - Research Support).

Privacy and security safeguards: Data set is pseudonymised. Each center will use the name of their city and case number. In case of any correction, update or more information, the responsible PI will be able to reach the patient and de-identify it to correct/ update the data. However, other centers will be blind, including us. Subjects will be registered in a key locked subject identification log, therefore no personal data will be shared. For the collaboration with the research partners, written agreements on data management, privacy and intellectual properties will be made. Data will not be transferred to any external parties. Data sharing agreement protect the rights of the research participants and data suppliers.

Data acquisition: Each center will collect the already recorded demographic, clinical, neuroimaging, biomarker, genetic and pathological data from their cohorts, retrospectively by using our guidebook. We will re-analyze the harmonized data. The data will not be reused.

Data collection: The data collection system is hosted by the ACA, namely CASTOR EDC (Electronic Data Capture EDC eConsent eCRF ePRO clinical research | Castor (castoredc.com)). A data definition (dictionary), data sharing agreement, the recommendations of our legal office, the platform for questions and open discussion, documents for ethical approval, data collection template, tutorials for MRI rating, guidebook will be available and will be stored in another safe electronic system, namely SURFdrive (SURFdrive: store and share your files securely in the cloud | SURF.nl) to be shared with our collaborators. Accounts and passwords have been established in both systems and data will be secured by password-protected system. Both electronic systems have an automatic track changes function which allows all collaborators to control the data.

Open data: Signed data sharing agreements between centers do not permit open data sharing.

AVAILABLE COGNITIVE TESTS IN THE COLLECTED DATASET							
**Memory**	**Language**	**Verbal semantic**	**Non-verbal semantic**	**Social cognition**	**Attention**	**Executive Functioning**	**Visuospatial**
Visual association task (VAT)-recall	Verbal agility	Boston Naming Test	CATS–Face matching	Mini-SEA	Digit span forward	Digit span backward	Fragmented letters (VOSP)
Visual episodic memory	Repetition	Warrington naming test	Benton famous faces	Ekman emotion recognition	Trail Making Test A	Trail Making Test B	Dot counting (VOSP)
Rey Auditory Verbal Learning Test (RAVLT) recall	Apraxia of speech rating	Benton Naming Test	Warrington famous faces	CATS Affect Matching	WMS R Digit Span Forward	WMS R Digit Span Backward	VOSP Silhouettes
Wechsler Memory Scale Logical Memory	Syntax Comprehension,	Pyramids and Palm Trees	Famous Faces Naming	The Awareness of Social Inference Test (TASIT) Emotion Evaluation		Symbol digit	VOSP object decision
Craft Story 21 Recall	Lexical fluency	VAT Naming	Famous Faces Familiarity	TASIT SI-M Sincere, Sarcastic		Weigls blocks	VOSP cube analysis
CERAD-Recall	Animal Fluency	Manchester Naming	Famous Faces Semantic Association	Revised Self-Monitoring Scale		Brixton errors	Benton line orientation
Short Story Recall	Letter fluency	Graded Naming	Famous Faces Name Familiarity	Interpersonal reactivity index		Brixton scaled score	
Free and Cued Selective Reminding			Famous Faces-Dutch	Interpersonal Adjective Scales			
California verbal memory				Emotional Theory of Mind			

Oktem delayed recall				Cognitive Theory of Mind			
ADAS delayed recall				Behavioral Inhibition BIS-total			
Manchester visual object memory				Behavioral Activation BAS fun/drive/reward			

Figure 1: Comparison of the centers. In order to avoid any potential selection bias, the mostly rated symptoms from 5 centers were compared.

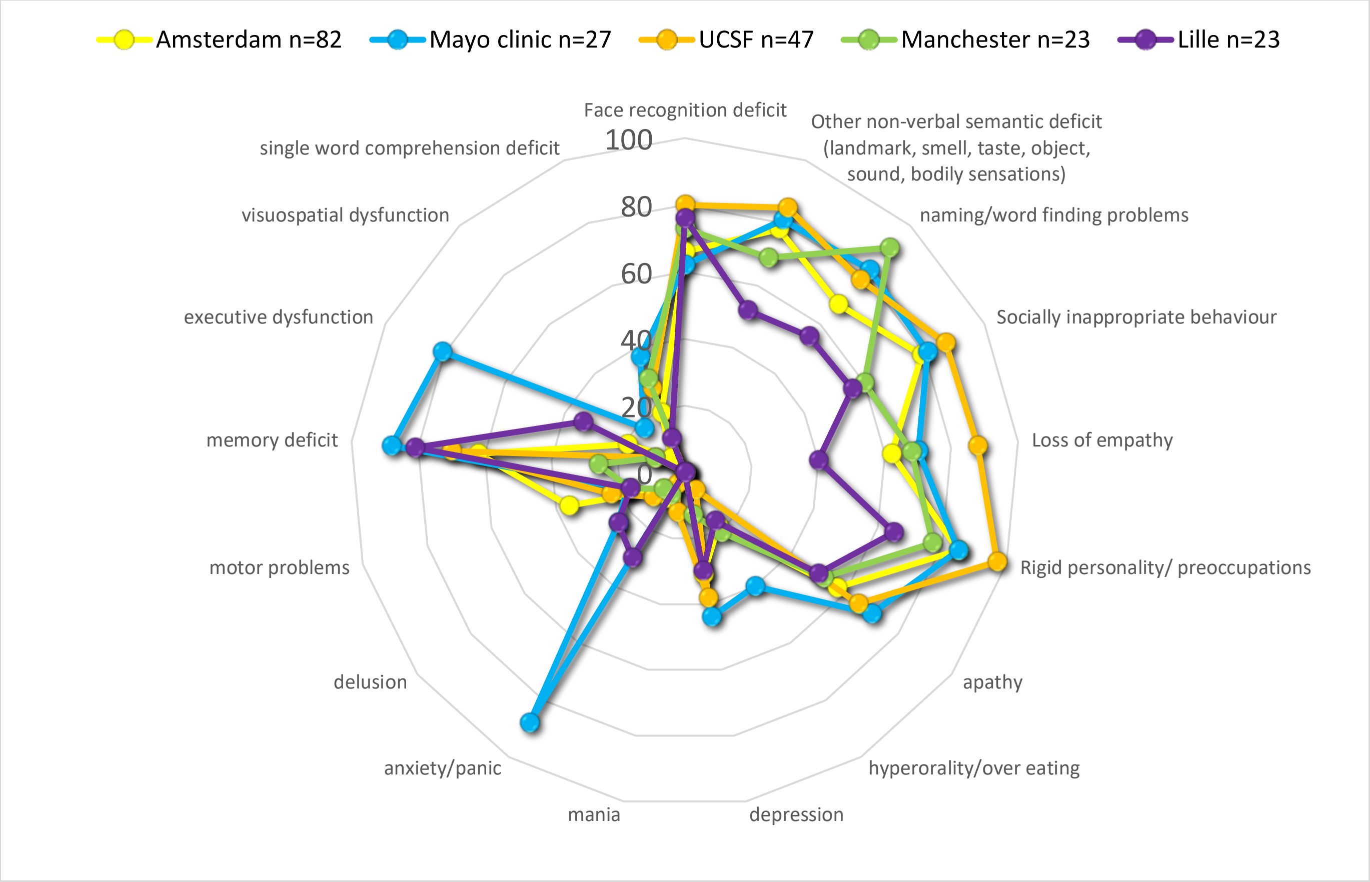

Figure 2: Comparison of the centers and total sample. To see whether the profile of total sample is representing centers, the results were also compared with the entire collected data

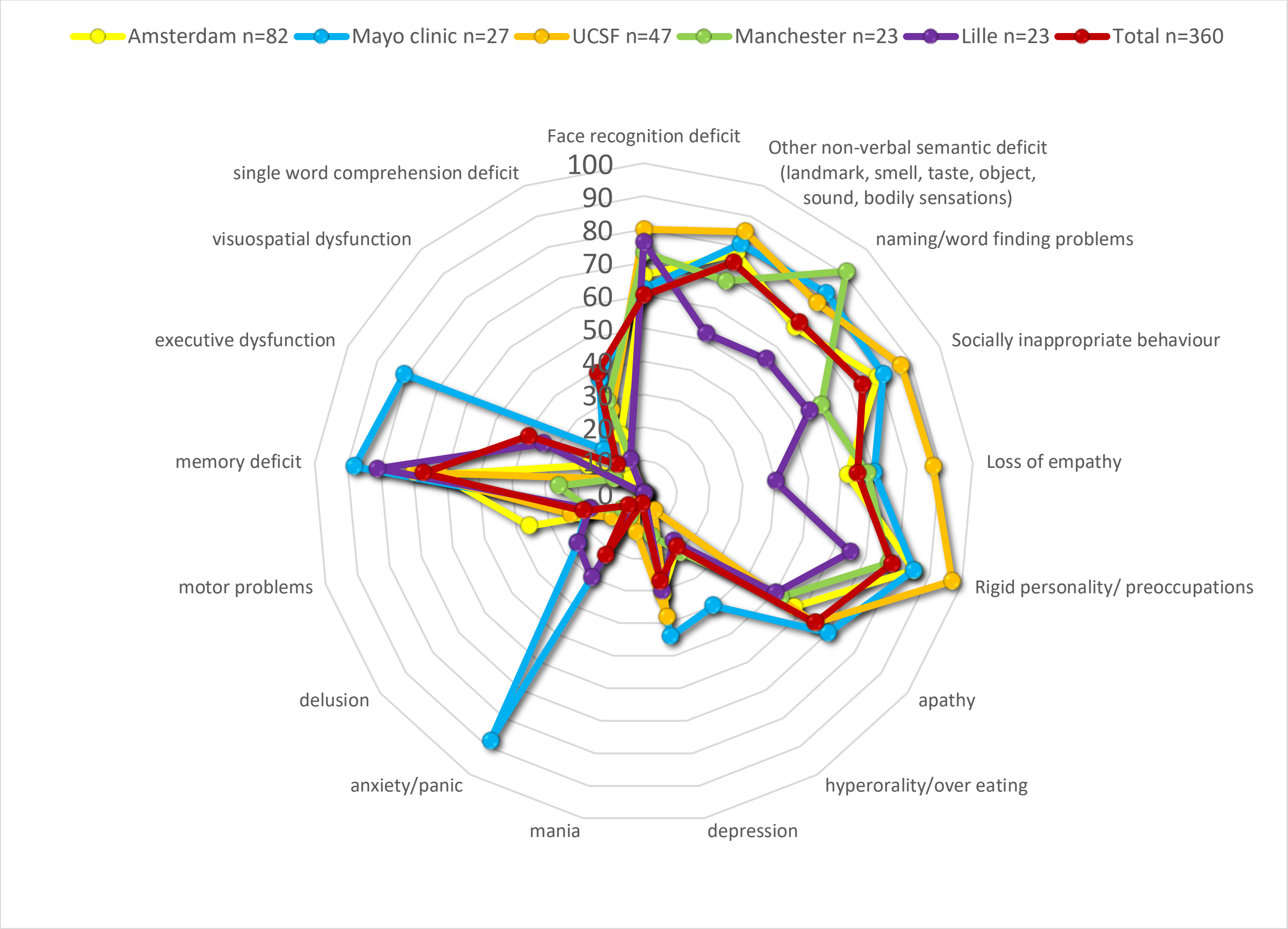

Supplement: Supplementary file 1 — Supporting Information [file ALZ-20-5647-s002.pdf]
